# Cardiac Arrhythmias in Muscular Dystrophies Associated with Emerinopathy and Laminopathy: A Cohort Study

**DOI:** 10.3390/jcm10040732

**Published:** 2021-02-12

**Authors:** Michał Marchel, Agnieszka Madej-Pilarczyk, Agata Tymińska, Roman Steckiewicz, Ewa Ostrowska, Julia Wysińska, Vincenzo Russo, Marcin Grabowski, Grzegorz Opolski

**Affiliations:** 1 1st Department of Cardiology, Medical University of Warsaw, 02-097 Warsaw, Poland; tyminska.agata@gmail.com (A.T.); r.steckiewicz@pro.onet.pl (R.S.); ewa.ostrowska713@gmail.com (E.O.); julia.wysinska@gmail.com (J.W.); marcin.grabowski@wum.edu.pl (M.G.); grzegorz.opolski@wum.edu.pl (G.O.); 2 Department of Medical Genetics, The Children’s Memorial Health Institute, 04-730 Warsaw, Poland; agamadpil@gmail.com; 3 Department of Translational Medical Sciences, University of Campania “Luigi Vanvitelli”—Monaldi Hospital, 80131 Naples, Italy; v.p.russo@libero.it

**Keywords:** Emery–Dreifuss muscular dystrophy, *LMNA*, *EMD*, emerin, lamin A/C

## Abstract

Introduction: Cardiac involvement in patients with muscular dystrophy associated with Lamin A/C mutations (*LMNA*) is characterized by atrioventricular conduction abnormalities and life-threatening cardiac arrhythmias. Little is known about cardiac involvement in patients with emerin mutation (*EMD*). The aim of our study was to describe and compare the prevalence and time distribution of cardiac arrhythmias at extended follow-up. Patients and methods: 45 consecutive patients affected by muscular dystrophy associated to laminopathy or emerinopathy were examined. All patients underwent clinical evaluation, 12-lead surface electrocardiogram (ECG), 24 h electrocardiographic monitoring, and cardiac implanted device interrogation. Results: At the end of 11 (5.0–16.6) years of follow-up, 89% of the patients showed cardiac arrhythmias. The most prevalent was atrial standstill (AS) (31%), followed by atrial fibrillation/flutter (AF/Afl) (29%) and ventricular tachycardia (22%). *EMD* patients presented more frequently AF/AFl compared to *LMNA* (50% vs. 20%, *p = 0.06*). Half of the *EMD* patients presented with AS, whilst there was no occurrence of such in the *LMNA* (*p* = 0.001). Ventricular arrhythmias were found in 60% of patients with laminopathy compared to 3% in patients with emerinopathy (*p* < 0.001). The age of AVB occurrence was higher in the *LMNA* group (32.8 +/− 10.6 vs. 25.1 +/− 9.1, *p* = 0.03). Conclusions: Atrial arrhythmias are common findings in patients with muscular dystrophy associated with *EMD*/*LMNA* mutations; however, they occurred earlier in *EMD* patients. Ventricular arrhythmias were very common (60%) in *LMNA* and occurred definitely earlier compared to the *EMD* group.

## 1. Introduction

Laminopathies and emerinopathies are genetic disorders caused by mutations in *LMNA* and *EMD* genes, respectively, encoding lamin A/C and emerin—ubiquitous proteins of the nuclear envelope. Both conditions show a heterogenous clinical presentation characterized by different neuromuscular and cardiac phenotypes. Cardiac involvement in patients with muscular dystrophy associated with laminopathy is typically characterized by atrioventricular conduction abnormalities, life-threatening cardiac arrhythmias, and heart remodeling towards dilated or restrictive cardiomyopathy [1]. Little is still known about cardiac involvement in patients with muscular dystrophy associated to emerinopathy. Additionally, data regarding timing of specific arrhythmias occurrence are still insufficient. The aim of our study was to describe and compare the prevalence and time distribution of cardiac arrhythmias in patients with muscular dystrophies associated with emerinopathy and laminopathy at extended follow-up.

## 2. Materials and Methods

### 2.1. Study Design and Population

This single-center prospective observational study included 45 consecutive patients affected by muscular dystrophy associated to laminopathy or emerinopathy admitted by and followed up at the 1st Department of Cardiology, Medical University of Warsaw. The study was approved by the Local Ethics Committee and was in accordance with the 1976 Declaration of Helsinki and its later amendments.

### 2.2. Study Protocol

Patients in the study underwent clinical evaluation, 12-lead surface electrocardiogram (ECG), 24 h electrocardiographic monitoring, cardiac implanted device interrogation at enrollment and every 12 months thereafter. Atrioventricular block (AVB) was assessed from a resting 12-lead electrocardiography (ECG) based on PR interval and P waves to QRS complexes relations. Atrial arrhythmias were collected from a resting ECG, Holter monitoring, pacemaker (PM), and implantable cardioverter-defibrillator (ICD) interrogation. Atrial arrhythmias were classified into supraventricular extra beats (SVEBs), atrial tachycardia (AT) with P wave of other than sinus morphology and rate 100–250/min, atrial flutter (AFl)—no P wave with F wave >250 min, and atrial fibrillation (AF)—no P wave with f wave >350/min. Atrial standstill (AS) or so-called atrial paralysis with no atrial activity was defined as no P, f, and F visible, confirmed by intracardiac electrograms. Nodal (junctional) rhythm (NR) was defined as a regular heart rate <50/min with narrow QRS complexes and no P waves preceding QRS complexes. Ventricular arrhythmias were classified as ventricular extra beats (VEBs), non-sustained ventricular tachycardia (nsVT), defined as ≥3 consecutive ventricular beats with a rate >120/min lasting <30 s, or sustained ventricular tachycardia (VT), defined as ventricular beats of a rate >120/min lasting >30 s.

### 2.3. Outcomes

The primary endpoint was the prevalence and the onset-time of cardiac arrhythmias among the study population.

### 2.4. Statistical Analysis

Distribution of continuous data was tested with the Kolmogorov–Smirnov and the Shapiro–Wilk test. Normally distributed variables were expressed as mean ± standard deviation (SD), whereas non-normal distributed ones as median (25th, 75th percentiles) and interquartile range (IQR). Categorical variables were reported as numbers and percentages. Differences between groups were compared using the Fisher exact test for categorical variables and the Mann–Whitney U test for continuous and ordinal variables. A two-sided *p*-value less than 0.05 was considered significant for all tests. All statistical analyses were performed using SPSS software, version 22 (IBM SPSS Statistics 22, New York, NY, USA).

## 3. Results

The baseline clinical, electrocardiographic, and echocardiographic characteristics of the study population are shown in Table 1. The study population included 30 patients with Emery–Dreifuss Muscular Dystrophy (EDMD1) (mutation in *EMD* gene encoding emerin) and 15 patients with muscular dystrophy associated with mutations in *LMNA* gene encoding lamin A/C: 12 patients with EDMD2, 2 with LGMD, and 1 with *LMNA*-related congenital muscular dystrophy (L-CMD). There were no significant differences in terms of baseline characteristics with the exception of gender, where 73% of *LMNA* patients were female and 80% of *EMD* were male (*p* < 0.001). All patients were free from other cardiovascular risk factors, which may be explained by their relatively young age. The median follow-up was 11 (5.0–16.6) years.

### 3.1. Cardiac Arrhythmias at Inclusion

**Figure 1:**
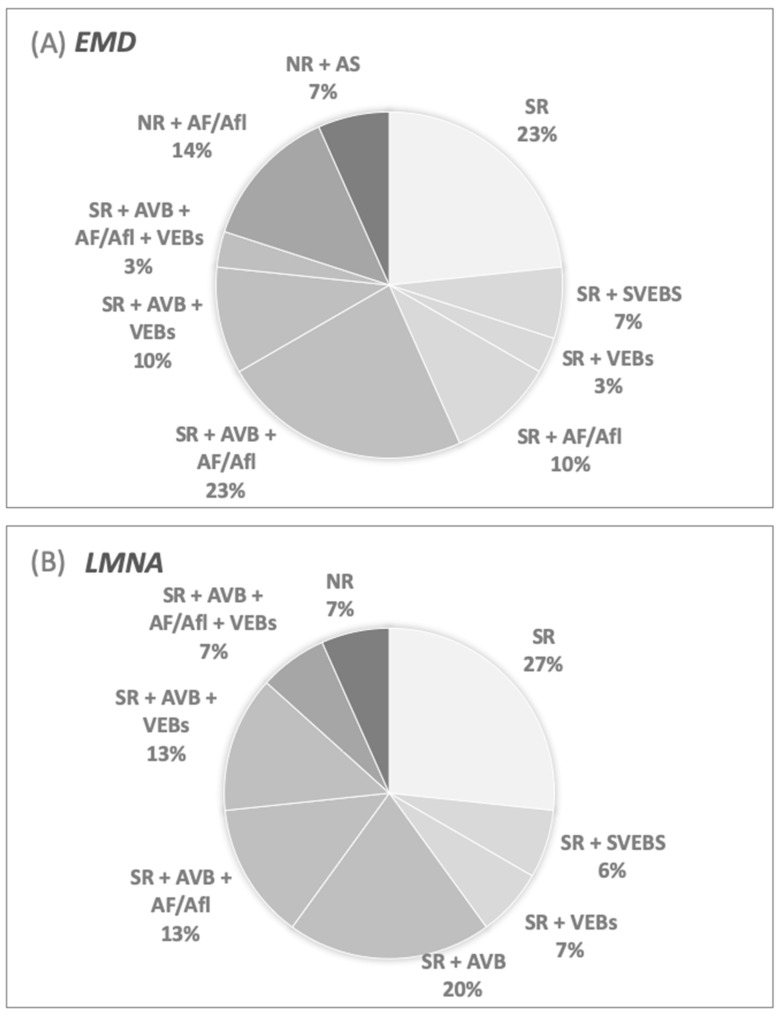
Cardiac arrhythmias at first evaluation. (**A**) Patients with mutation in *EMD* gene. (**B**) Patients with mutation in *LMNA* gene. AF—atrial fibrillation; AFl—A=atrial flutter; AS—atrial standstill; AVB—atrio-ventricular block; *EMD*—mutation in *EMD* gene; *LMNA*—mutation in *LMNA* gene; NR—noDAL RHYTHM; SR—SINUS rhythm; SVEBs—supraventricular extra beats; VEBs—ventricular extra beats.

The mean age of the study population at first cardiac evaluation was 24.9 +/− 12 years. At the first electrocardiographic evaluation, 84% (*n*: 38) of patients showed sinus rhythm and 16% (*n*: 7) junctional rhythm. This was more common in the *EMD* group, although the difference was not statistically significant (20% vs. 6.7%; *p* = 0.4). Forty percent (*n*: 18) experienced AF or AFl at the first evaluation. An increasing trend in AF/AFl prevalence in the *EMD* group was shown (50% vs. 20%, *p* = 0.06). AS was shown in 7% of the study population (*n*: 3), and all of them were patients with emerinopathy. AVB were present in 58% of the study population, in particular the first-degree AVB in 11% (*n*: 5), second-degree AVB in 24% (*n*: 11), and third-degree in 22% (*n*: 10). No significant differences between *EMD* and *LMNA* were found (Table 2). In 13% of patients (6/45) VEBs were present in Holter monitoring, wherein 2/30 were from the *EMD* group and 4/15 from the *LMNA* group, respectively. No patients presented nsVT or VT at initial evaluation. The occurrence of different arrhythmias and conduction disturbances at first cardiac evaluation are presented in Table 2 and Figure 1.

### 3.2. Prevalence of Atrial Arrhythmias at Follow-Up

The mean age at the end of follow-up was 36.3 +/− 14.4 years. The prevalence of atrial arrhythmias at the end of follow-up is presented in Table 3. Only 22% of patients (*n*: 10) remained free from the sustained supraventricular arrhythmias or AS. One-third of patients with laminopathy and one-fourth with emerinopathy had AF/AFl. Almost half of the *EMD* group (*n*: 14) presented with AS, which did not occur in any patient from the *LMNA* group (*p* 0.001). Seventy-six percent of *EMD* patients needed PM implantation, while the percentage of PM implantation in the *LMNA* group was 47% (*p* 0.09). Half of the patients presented with SVEBS. The details are shown in Table 3.

### 3.3. Prevalence of Ventricular Arrhythmias at Follow-Up

One-fourth of patients presented with nsVT—a potential risk factor for sudden cardiac death—and had an implantable cardioverter-defibrillator (ICD) implanted. Apart from ventricular arrhythmias being more frequent in laminopathies, more differences in terms of ventricular arrhythmias between patients from the *EMD* and *LMNA* groups were observed. nsVT was present in as much as 60% (*n*: 9) of *LMNA* patients, while only 3% of *EMD* patients (*n*: 1) had nsVT (*p* < 0.001). Moreover, premature ventricular complexes (PVCs) or PVC couplets (considered as more benign arrhythmias), already present at initial evaluation, finally occurred in 60% (*n*: 9) of patients with laminopathy and in 30% (*n*: 9) of patients with emerinopathy. Since ventricular arrhythmias occurred more frequently in the *LMNA* group, the number of implanted ICD devices was accordingly higher in these patients (*p* < 0.001). Ventricular arrhythmias occurrence is shown in Table 3.

### 3.4. Timing of Arrhythmia’s Occurrence

**Figure 2:**
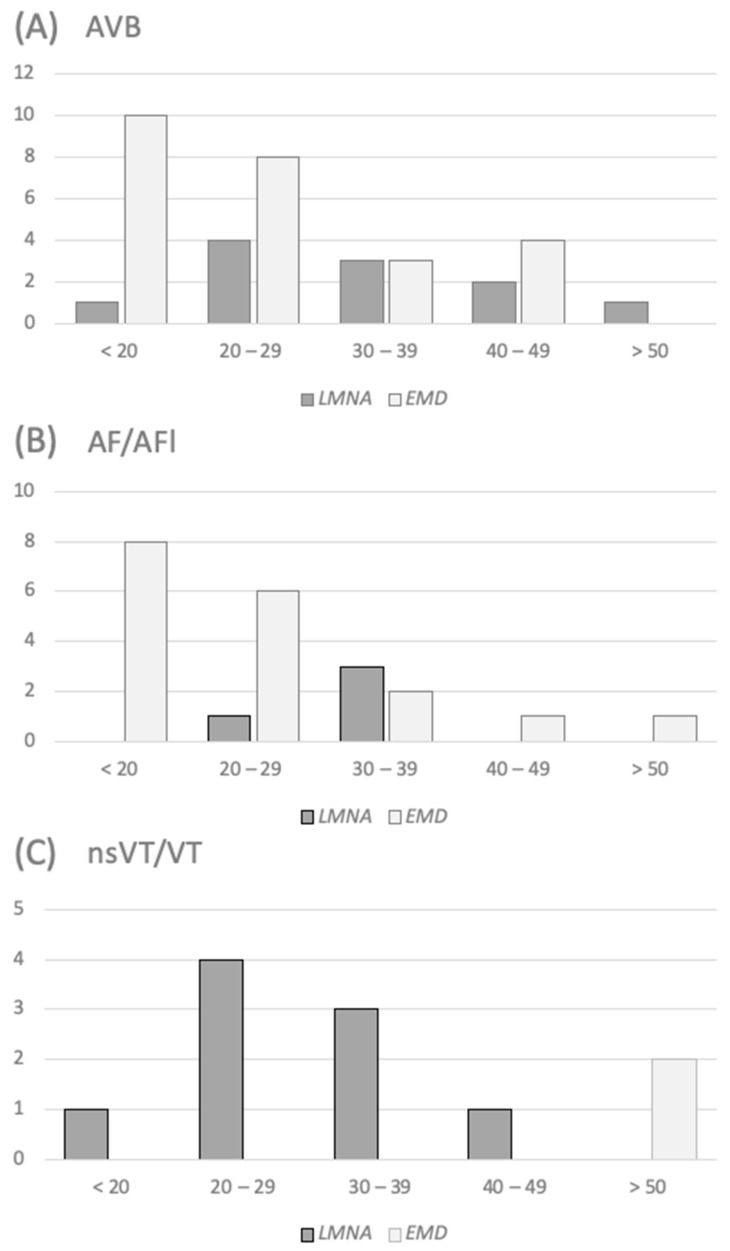
Age distribution of the occurrence of cardiac arrhythmias. (**A**) AVB – atrio-ventricular block. (**B**) AF/AFl Atrial fibrillation and atrial flutter. (**C**) nsVT/VT Non-sustained ventricular tachycardia and ventricular tachycardia. AF—atrial fibrillation; AFl—atrial flutter; AVB—atrio-ventricular block; EMD—mutation in EMD gene; LMNA—mutation in LMNA gene; nsVT—non-sustained ventricular tachycardia; VT—ventricular tachycardia.

**Figure 3:**
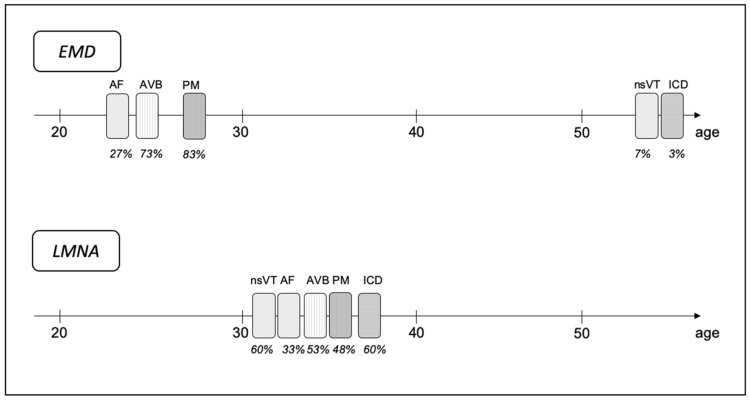
Timeline of arrhythmic events and interventions for both *EMD* and *LMNA* patients. The position of the bar on the line corresponds to the mean age of event occurrence. The percentage below shows the frequency of the event. AF—atrial fibrillation; AVB—atrio-ventricular block; *EMD*—mutation in *EMD* gene; ICD—implantable cardioverter-defibrillator; *LMNA*—mutation in *LMNA* gene; nsVT—non-sustained ventricular tachycardia; PM—pacemaker.

Furthermore, the time of the arrhythmia’s occurrence was analyzed, including the evaluation on the differences between *EMD* and *LMNA* cohorts, and are depicted in Figure 2 and Figure 3. In our group, the age of AVB occurrence was relatively higher in patients with laminopathy (32.8 +/− 10.6 vs. 25.1 +/− 9.1, *p* = 0.03). Difference in the age of AF/AFl onset (31.8 +/− 3.9 vs. 24.2 +/− 10.4, *p* = 0.053) for *LMNA* and *EMD* patients, respectively, was close to significant. As for ventricular arrhythmias, patients with emerinopathy were generally older at the time of first occurrence, although the differences did not reach statistical significance.

In all patients with emerinopathy evident clinical signs of skeletal muscle involvement, typically seen in the first decade of life, preceded cardiac symptoms, which occurred at the end of the second decade or slightly later. However, this was not true for patients with laminopathy, as in some of them cardiac arrhythmia was the first health problem, being the reason to seek medical advice. Only further detailed neurological assessment led to final diagnosis of skeletal muscle laminopathy.

## 4. Discussion

Several muscular dystrophies manifest in cardiac involvement. Knowledge of the incidence and timeline of occurrence of different arrhythmias may be crucial for cardiac screening, as well as thromboembolic and SCD risk assessment. In the most common X-linked muscular dystrophinopathies, Duchenne (DMD) and Becker (BMD) muscular dystrophy, dilated cardiomyopathy (DCM) precedes appearance of severe cardiac arrhythmias in a typical scenario. The time course of cardiac dysfunction in DMD is fairly well predictable [2]. Muscular dystrophies associated with laminopathies belong to the group of ultra-rare diseases (incidence of 0.39 per 100,000) [3]. Thus, the natural course of arrhythmias is more difficult to establish due to smaller groups of patients available for observation. More and more studies concerning *LMNA*-positive patients are being conducted [4,5]. In the muscular dystrophies due to laminopathies the risk of arrhythmias increases with age (the penetrance of *LMNA* mutations is almost complete for cardiac phenotype), but their occurrence may be different in patients with emerinopathy and laminopathy. Early cardiac involvement in laminopathies is usually characterized by a prolonged PR interval, which may progress to advanced AVB and is explained by gradual replacement of myocardium by fibrous and adipose tissue [6,7]. Several hypotheses have been proposed to explain the problem of cardiac phenotype variability in patients with mutated *LMNA* or *EMD* genes [8,9]. It has been suggested that in *EMD* peripheral muscle manifestation usually occurs before cardiac symptoms [9]. There is a fair amount of data concerning differences in peripheral muscle involvement including the results from microscopic examinations of the muscle biopsies [10,11]. In addition, previous papers suggested that patients with laminopathy and neuromuscular presentation had an earlier and more advanced cardiac involvement [12,13]. However, none provided a direct comparison of cardiac involvement in *EMD* and *LMNA* in a considerable cohort and extended follow-up yet.

In our study atrioventricular conduction abnormalities typical for laminopathies were already present at the first cardiac screening. As many as 14% of patients had junctional escape rhythm in ECG tracings. According to guidelines, in patients with muscular dystrophy associated with *EMD* mutations, early implantation of the PM may be justified, while in laminopathy, primary prevention of SCD should be realized by ICD implantation [14]. In 44% of our cohort first cardiac evaluation ends up with the decision of PM implantation. Interestingly, during follow-up, AVB occurred significantly earlier in patients with emerinopathy (usually in the second or third decade of life), while the time of its distribution in laminopathy was more spread over the decades (Figure 2 and Figure 3). This discovery might be consistent with the findings of Hong et al. [15]. They described three cases, among them two *EMD* and one *LMNA* patient presented with AS and junctional nodal rhythm, although the *LMNA* patient was in the fourth decade, while *EMD* patients were in the second decade of their lives. In a cohort of 79 Norwegian *LMNA* DCM patients and asymptomatic family members, 72% presented with AVB and 37% were PM-dependent at the end of the follow-up [16]. The need for pacing in EDMD patients was previously described in a paper by Steckiewicz et al. [17], where most of the patients were implanted with a PM and one with an ICD. Of 41 of patients with laminopathy and skeletal muscle involvement described in the paper by Bonne et al., 23 had arrhythmias, 6 were implanted with a PM, and 1 with an ICD, but no more details were provided [10]. In van Berlo’s meta-analysis 28% of patients with laminopathy received a PM [4]. Little is known about cardiac resynchronization therapy (CRT) applied in EDMD patients. In one of the biggest cohorts of *LMNA* patients with a neuromuscular onset [12] only two patients were implanted with CRT-D in primary prevention. In our group only one patient with *LMNA*, low ejection fraction, and symptomatic heart failure was implanted with CRT-D. These low numbers are probably due to preserved systolic function and no signs of heart failure at the time of the occurrence of severe atrio-ventricular conduction disturbances. This is particularly true for *EMD*. In *LMNA* there is probably more room for CRT, which should be considered in patients with signs of cardiomyopathy, especially when ICD is needed for SCD prevention and the CRT-D device may be implanted.

Several longitudinal studies suggested that AF and AFl are the most frequent cardiac arrhythmias in laminopathies [18]. Many patients at first cardiac evaluation already had supraventricular arrythmias present, which may suggest that they are low-symptomatic at early stage of the disease [17]. On the contrary, especially in case of AF and AFl, young patients in the general population usually develop symptoms of arrhythmia. In the *EMD* group the onset of AF and AFl occurred in the second or third decade of life in the majority of patients. This is uncommon for any other muscular dystrophy. In the *LMNA* group the prevalence of this arrhythmia was less frequent yet still significant, and the mean age of the occurrence was higher in comparison to the *EMD* group. Nevertheless, the patients were relatively young (mean age 32). Realizing the early occurrence of asymptomatic AF patients with emerinopathy and laminopathy may be of great importance due to elevated thromboembolic risk (even without symptoms of AF) and emphasizes the necessity of early cardiac screening. The AS phenomenon, described as pathognomonic for *EMD* patients [18,19], was present at the first screening in only 7% of our patients. Interestingly, at the end of follow-up one-third of patients from the *LMNA* group developed AF, one-third AT, yet no patient had AS. In the *EDM* group the prevalence of supraventricular arrhythmias was even higher with half of the patients with AS and one-fourth with AF/AFl, all together 10% presented with AT. The mean age when AS had been confirmed was 34 years. The third and fourth decade of life used to be considered typical for the onset of atrial arrythmias in EDMD. In a paper by Bialer et al. affected patients younger than 20 years old did not present any ECG changes, while all affected men at the age of 35 years or older already had arrhythmias [20]. Boriani et al. [21] described 18 EDMD patients, both with emerinopathy (10) and laminopathy (8). Sixty-one percent (*n*: 11) experienced AF/Afl during follow-up. Forty-five percent (*n*: 5) of those who had AF/AFl subsequently developed AS. AF/AFl were present in both *EMD* and *LMNA* groups, irrespectively of severity of muscle involvement. In our group 78% of all EDMD patients presented either AF, AFl, AT, or AS at the end of follow-up. Our research suggests a significant difference in the time of occurrence of atrial arrhythmias in laminopathic patients with different genetic background.

Patients with laminopathies are at risk of SCD. This phenomenon is present in *LMNA* patients with both EDMD2, LGMD, and pure DCM without any peripheral muscle involvement [1,22,23]. There are dedicated risk calculators to assess the SCD risk in *LMNA*-positive subjects [24,25]. Several risk factors for malignant ventricular arrhythmias have also been identified. According to van Rijsingen et al. [26] the following four are the most important ones: male gender, nonsense mutation (ins- del/truncating or mutations affecting splicing), left ventricle ejection fraction (LVEF) <45% at first medical contact, and presence of nsVT. Among our cohort of 15 *LMNA* patients there were 4 male patients, 2 with non-sense mutation, 5 with decreased LVEF (<45%), and 9 patients presented with nsVT. This translates into five *LMNA* patients with no risk factors, four with one, and six with two or more. One *LMNA* and one *EMD* patient were implanted with ICD in secondary prevention, while the other eight *LMNA* in primary prevention. Although VT in patients with *EMD* mutation who had ICD implanted was previously described [27,28], the frequency of this form of arrhythmia in emerinopathy is not fully defined. The only one *EMD* patient implanted with ICD in our cohort had a reduced LVEF, while in *LMNA* ventricular arrythmias were present in patients with both reduced (5/15) and preserved LVEF (4/15). This may be an argument for a thesis that ventricular arrhythmias may precede systolic dysfunction in the *LMNA* group, which was not described in the *EMD* group. In patients from the *LMNA* group ventricular arrhythmias occurred in the third and fourth decade of life, while in the *EMD* group it was postponed above age fifty. One-fourth of all *LMNA* patients with muscular involvement presented significant ventricular arrhythmias, although in the laminopathy subgroup its prevalence was as high as 60%.

Limitation of the study. Follow-up depended on patients’ age at first presentation. Some patients have been available since childhood, while others had their first consultation in adulthood. Therefore, the precise determination of the onset, type and severity of skeletal muscle symptoms, and sequence of cardiac and muscle involvement were difficult to establish. Frequency of the follow-up was not the same in all patients due to various adherence to medical recommendations resulting from disability and social circumstances.

The purpose of the current study was to analyze the occurrence of arrythmias in a Polish cohort of patients with laminopathy and emerinopathy, both coexisting with peripheral muscle involvement. Atrial arrhythmias were the most common arrhythmia in this group. In the *EMD* group it occurred first usually in the second or third decade of life. In the *LMNA* group it seemed to occur later. At the end of follow-up only 22% of patients were free of either AF, Afl, AT, or AS. AS did not occur in the patients with *LMNA* mutation from our cohort. Many patients with laminopathies presented with AVB early in the course of the disease. In *EMD* patients it occurred significantly earlier than in *LMNA* patients, in whom time distribution was more spread over the years. Two-thirds of patients ended up with a pacemaker at the end of follow-up. Ventricular arrhythmias were very common among patients from the *LMNA* group and occurred definitely earlier compared to the patients from the *EMD* group, whereas no significant ventricular arrhythmias occurred before the age of 50. The difference in cardiac arrhythmias occurrence in *LMNA* and *EMD* groups indicates a need for precise genetic diagnosis amongst patients with muscular dystrophy. On the other hand, atrioventricular conduction abnormalities and/or early onset of atrial arrhythmia may be a red flag to search for laminopathy in otherwise healthy young patients without any known previous neurologic diagnosis [29,30]. Does arrythmia burden in neuromuscular dystrophies contribute to the risk of clinical events? This is a question for future research.

## Figures and Tables

**Table 1:** Baseline characteristics of the study population.

	*EMD* Group *n* = 30	*LMNA* Group *n* = 15	*p*-Value
Age (years)	21.0 (15.25–30.0)	26.0 (18.0–33.0)	NS
Female (%)	20	73	<0.001
BMI (kg/m^2^)	21.5 (19.4–25.2)	20.2 (17.3–25.1)	NS
Sporadic/familial	13/17	9/6	NS
LVEDV (mL)	119 (90–169)	103 (86–125)	NS
LAV (mL)	56.4 (47.8–73.3)	50.5 (38.5–63)	0.08
LVEF (%)	52 (48–58)	54 (48–58)	NS
NTpro-BNP (pg/mL)	70 (44–102)	109 (54–347)	NS
NYHA I-II (%)	0	20	0.08
NYHA III-IV	0	0	NS

BMI—body mass index; EMD—mutation in EMD gene; LAV—left atrial volume; LVEDV—left ventricle end-diastolic volume; LVEF—left ventricle ejection fraction; LMNA—mutation in LMNA gene; NS – not significant; NTpro-BNP—N-terminal pro hormone B-type natriuretic peptide; NYHA—New York Heart Association class.

**Table 2:** Differences in arrhythmias occurrence at initial evaluation.

	Total Group (*n* = 45)	*EMD* Group (*n* = 30)	*LMNA* Group (*n* = 15)	*p*-Value
SR, % (*n*)	84.4 (38)	80 (24)	93 (14)	0.40
NR, % (*n*)	15.6 (7)	20 (6)	6.7 (1)	0.40
AT, % (*n*)	24.4 (11)	16.7 (5)	40 (6)	0.14
AF, % (*n*)	28.9 (13)	33.3 (10)	20 (3)	0.49
AFl, % (*n*)	17.8 (8)	23.3 (7)	6.7 (1)	0.24
AS, % (*n*)	6.7 (3)	10 (3)	0 (0)	0.54
AVB 1st degree, % (*n*)	11.1 (5)	10 (3)	13.3 (2)	1.00
AVB 2nd degree, % (*n*)	24.4 (11)	20 (6)	33.3 (5)	0.46
AVB 3rd degree, % (*n*)	22.2 (10)	26.7 (8)	13.3 (2)	0.46
SVEBs, % (*n*)	37.8 (17)	36.7 (11)	40 (6)	1.00
VEBs, % (*n*)	13.3 (6)	6.7 (2)	26.7 (4)	0.16
nsVT, % (*n*)	0 (0)	0 (0)	0 (0)	-
VT, % (*n*)	0 (0)	0 (0)	0 (0)	-

AF—atrial fibrillation; AFl—atrial flutter; AS—atrial standstill; AT—atrial tachycardia; AVB—atrio-ventricular block; *EMD*—mutation in *EMD* gene; *LMNA*—mutation in *LMNA* gene; NR—nodal rhythm; nsVT—non-sustained ventricular tachycardia; SR—sinus rhythm; SVEBs—supraventricular extra beats; VEBs—ventricular extra beats; VT—ventricular tachycardia.

**Table 3:** Occurrence of atrial and ventricular arrhythmias at the end of follow-up.

	Total Group (*n* = 45)	*EMD* (*n* = 30)	*LMNA* (*n* = 15)	*p*-Value
SVEBs, % (*n*)	48.9 (22)	46.7 (14)	53.3 (8)	0.76
AT only, % (*n*)	17.8 (8)	10 (3)	33.3 (5)	0.10
AF/Afl only, % (*n*)	28.9 (13)	26.7 (8)	33.3 (5)	0.73
AS only, % (*n*)	31.1 (14)	46.7 (14)	0 (0)	0.001
No AT/AF/AFl/AS, % (*n*)	22.2 (10)	16.7 (5)	33.3 (5)	0.26
VEBs, % (*n*)	40 (18)	30 (9)	60 (9)	0.11
VEBs couplets, % (*n*)	22.2 (10)	13.3 (4)	40 (6)	0.06
nsVT, % (*n*)	22.2 (10)	3.3 (1)	60 (9)	<0.001
VT, % (*n*)	8.9 (4)	6.7 (2)	13.3 (2)	0.59
nsVT/VT, % (*n*)	24.4 (11)	6.7 (2)	60 (9)	<0.001
PM implantation, % (*n*)	66.7 (30)	76.7 (23)	46.7 (7)	0.09
ICD implantation, % (*n*)	22,2 (10)	3,3 (1)	60 (9)	<0.001

AF—atrial fibrillation; AFl—atrial flutter; AS—atrial standstill; AT—atrial tachycardia; *EMD*—mutation in *EMD* gene; ICD—implantable cardioverter-defibrillator; *LMNA*—mutation in *LMNA* gene; nsVT—non-sustained ventricular tachycardia; PM—pacemaker; SVEBs—supraventricular extra beats; VEBs—ventricular extra beats; VT—ventricular tachycardia.

## Data Availability

The data presented in this study are available on request from the corresponding author. The data are not publicly available in order to protect patient privacy.
